# Gestational and Type 2 Diabetes in Relation to Urinary Incontinence in Black Women in the U.S.

**DOI:** 10.3390/uro5020008

**Published:** 2025-04-08

**Authors:** Yvette C. Cozier, Nelsy Castro-Webb, Kimberly A. Bertrand, Miatta Ndama, Toby C. Chai, Bernard L. Harlow, Padmasini Kandadai, Shanshan Sheehy, Lynn Rosenberg

**Affiliations:** 1 Slone Epidemiology Center, Boston University, 72 E. Concord Street, Boston, MA 02118, USA; 2 Department of Epidemiology, Boston University School of Public Health, 715 Albany Street, Boston, MA 02118, USA; 3 Department of Medicine, Boston University Chobanian & Avedisian School of Medicine, Boston, MA 02118, USA; 4 Department of Urology, Boston University Chobanian & Avedisian School of Medicine, 72 E. Concord Street, Boston, MA 02118, USA; 5 Department of Obstetrics and Gynecology, Boston University Chobanian & Avedisian School of Medicine, 85 E. Concord Street, Boston, MA 02118, USA

**Keywords:** urinary incontinence, gestational diabetes, type 2 diabetes, black women, urge incontinence, stress incontinence

## Abstract

**Background/Objectives:**

Urinary incontinence (UI) is a common condition in women of all ages. Type 2 diabetes (T2D) has been associated with UI, but gestational diabetes (GD), glucose intolerance first recognized during pregnancy, has received relatively little attention as an independent risk factor for UI. We explored the roles of GD and T2D, independently and in combination, on the risk of UI in a Black Women’s Health Study (BWHS), a follow-up of Black women in the U.S. aged 21–69 at enrolment in 1995.

**Methods:**

We analyzed the 28,978 parous women who had information on GD, T2D, and UI in 2011. We estimated odds ratios (OR) and 95% confidence intervals (95% CI) using logistic regression with adjustment for several important variables, including age, parity, body mass index, and diuretic use. We also stratified analyses according to T2D status (T2D, no T2D).

**Results:**

The multivariable-adjusted ORs for women with a history of GD, compared to those without, was 1.18 (95% CI: 1.02, 1.37), for UI frequency of ≥1/week; the estimate among women with a history of T2D, compared to those without, was 1.16 (1.06, 1.27) for the same frequency. In stratified analyses, GD was associated with a 23% increased risk of weekly UI among women without a history of T2D, while there was no association observed among those with a history of T2D.

**Conclusions:**

In the BWHS, GD was positively associated with urinary incontinence, independent of T2D status. Our results suggest that women who experience GD—even without subsequent development of T2D—might be at increased risk of UI and may benefit from early intervention.

## 1. Introduction

Urinary incontinence (UI), defined as any involuntary leakage of urine, is a common condition in women of all ages (1–3). In the U.S., the reported prevalence of UI among adult women is 62%, with nearly a third reporting symptoms at least monthly (4). Global estimates range from 5% to 70%, and prevalence increases with age (3, 5). UI is routinely classified into three subtypes: stress (loss of urine upon exertion, including coughing, lifting, or laughing), urgency (loss of urine with a strong need to urinate), and mixed (co-existing symptoms of stress and urgency), with stress UI being the most common (4, 6). UI is associated with embarrassment, decreased participation in social/physical activity (7), and disruption of daily life (8). Direct costs for treatments, extra laundry, absorbent pads, and diapers have been estimated to exceed $12 billion/year (9), while the estimated societal costs (lost wages, quality of life) are estimated to exceed $80 billion annually (10). Some studies have reported increased frequency of UI among Black women compared with non-Hispanic White (NHW) women and Asian women (6, 11–13), while others report decreased UI among Black women, including severe UI (wetting of underwear) (14) compared to NHW women (15–17).

Type 2 diabetes (T2D) has been associated with UI (14, 16, 18–23) and is estimated to increase the risk of UI more than twofold (24). Gestational diabetes (GD) is a state of glucose intolerance first recognized during pregnancy (25, 26) and accounts for over 90% of all cases of diabetes in pregnancy (27). Glucose levels often return to pre-pregnancy levels during the postpartum period, but affected women have an increased risk of developing T2D in the future compared to non-affected women (18, 19, 28). In 2020, the overall prevalence of GD was highest for non-Hispanic Asian women (14.9%) and lowest for Black (7.0%) and White (6.5%) women; the prevalence for Hispanic women was 8.5% (29–31). Black women with a history of GD, however, are more likely to develop T2D than White women with a history of GD (32, 33).

GD has received relatively little attention as an independent risk factor for UI. Both animal and human studies suggest that the hypoglycemic environment may impair the structure, morphology, and function of skeletal muscle tissue, including the pelvic floor (34, 35). Studies in the US (36), Taiwan (37), and Brazil (38, 39) have reported positive associations between GD and UI during pregnancy (36) and within two years of a term delivery (37–39). Yet other studies have linked pre-diabetes, an intermediate phase of blood glucose dysregulation (40), with both stress and urgency UI (14, 20). Studies of both T2D and GD, to date, have included few or no Black/African American women (20, 21, 36, 37). We therefore sought to explore the role of GD and T2D on the risk of UI (including UI subtypes) in a cohort of American Black women. We also assessed the role of GD alone and in combination with T2D, on UI risk.

## 2. Materials and Methods

### The Black Women’s Health Study (BWHS).

In 1995, 64,500 Black women ages 21–69 years (median 38 years) from the continental U.S. enrolled in the BWHS by completing a 14-page health questionnaire (41–43); the 59,000 women who completed the first (1997) and/or second (1999) follow-up questionnaires, in addition to the baseline (1995) questionnaire, comprise the cohort that has been followed. At baseline, participants provided data on demographics, anthropometry (current weight and height, weight at age 18), medical and reproductive history, vigorous physical activity, cigarette smoking, alcohol use, and other variables. Inclusion in the cohort was independent of comorbid illness(es). Biennial follow-up questionnaires and yearly linkage with the National Death Index provide updated information. Follow-up of the cohort has been successful for >80% of potential person-years. The Institutional Review Board of Boston University Medical Campus approved the study and participants have indicated their consent by filling out and returning study questionnaires.

**Figure 1.**
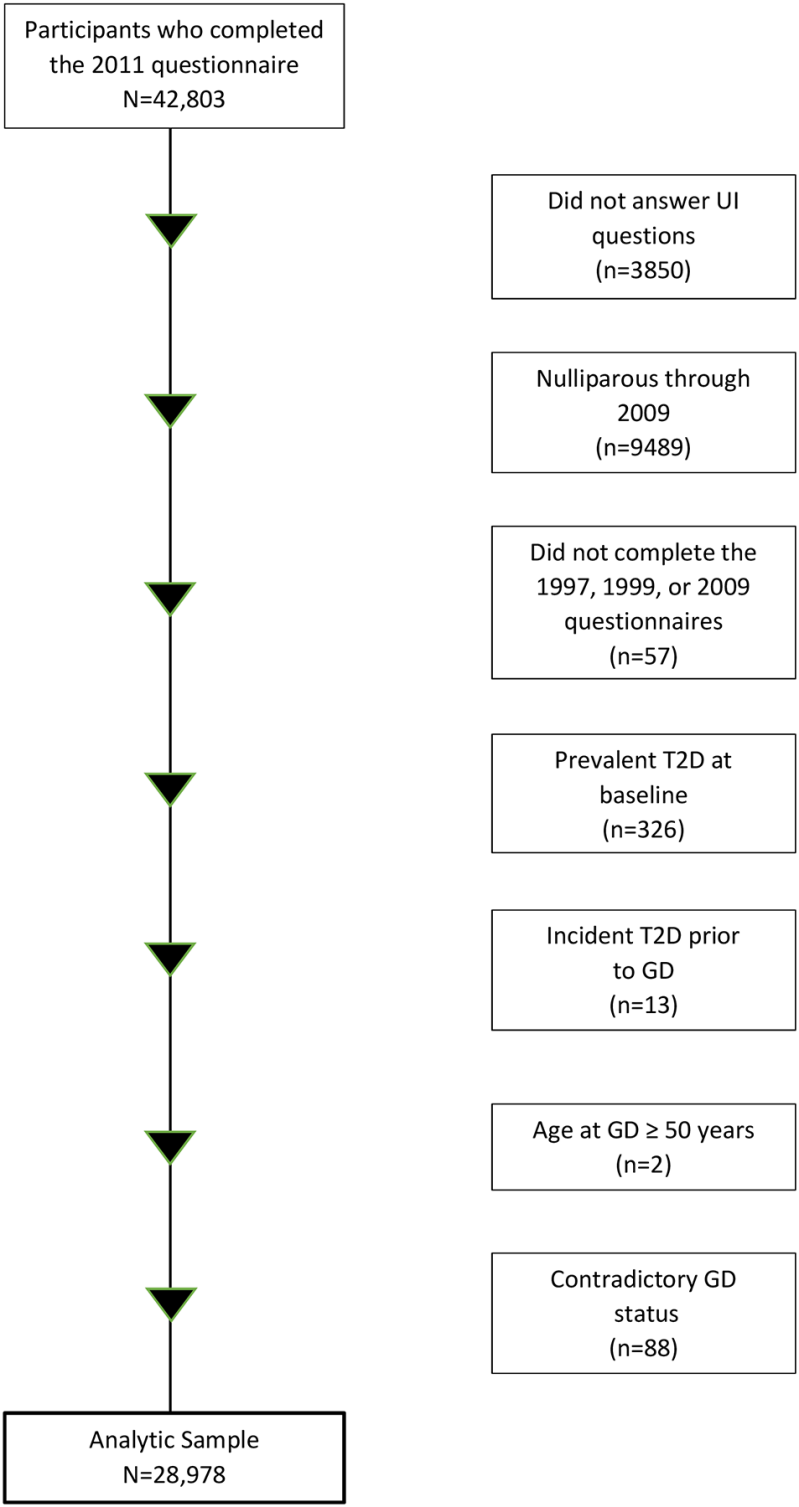
BWHS flow chart of gestational diabetes (GD) and urinary incontinence (UI), 2011.

Potential participants for the current analysis were the 42,803 women who completed the 2011 questionnaire, which included questions about UI. From these women, we excluded those who did not answer the UI questions (n = 3850); remained nulliparous through 2009 (n = 9489); did not complete the 1997, 1999, or 2009 questionnaires (which asked about GD) (n = 57); reported prevalent diabetes at baseline in 1995 (n = 326); reported incident diabetes prior to GD (n = 13); whose age at GD was ≥50 years (n = 2); or gave contradictory reports of GD status (n = 88), leaving an analytic sample of 28,978 (Figure 1).

#### Gestational Diabetes (GD).

The 1997, 1999, and 2009 questionnaires asked questions about the history of GD. The 1997 questionnaire asked, “If a doctor has told you that you had any…” of a list of conditions, including “diabetes during pregnancy” and whether diagnosis occurred before or after “1 March 1995”, the start of the BWHS. The 1999 questionnaire asked whether “Between March 1997 and March 1999, you were diagnosed with diabetes during pregnancy”, with space for the participant to provide the year of diagnosis. The 2009 questionnaire asked, “Did you ever develop diabetes during a pregnancy (gestational diabetes)?” Response options included “no”, “yes” (if yes, “how old were you?”), and “don’t know”.

We assessed the reproducibility of the GD variable in a sample of BWHS participants. During each follow-up cycle, multiple waves of questionnaires are mailed to women who have not yet responded. During the 2009 follow-up cycle, when the GD questions were most recently asked, 1319 women returned duplicate questionnaires. There was 90% agreement between the first and duplicate questionnaires for those reporting a diagnosis of GD.

#### Type 2 Diabetes (T2D).

The baseline and all follow-up questionnaires ask specifically about the diagnosis of T2D, as well as the date of diagnosis. We defined T2D as a report of diabetes at age 30 or older. In a BWHS validation study, 293 women reported a diagnosis of incident T2D during follow-up and provided permission to contact their physicians. A completed physician checklist was returned for 229 participants; a diagnosis of diabetes was confirmed for 220 (96%) (44). The estimated prevalence of undiagnosed T2D in the BWHS was 6.1% based on tests for hemoglobin A1C (HbA1C) (≥6.5%) among 10,249 participants who provided a blood sample to the BWHS but had never previously reported T2D (45).

#### Urinary Incontinence (UI).

Questions on UI were included for the first time on the 2011 questionnaire. Two questionnaire items asked about the frequency of UI in the past year and the cause (type) of leakage. The question about the frequency of UI asked, “During the past year, how often have you leaked or lost control of your urine?” Response options were never, less than once per month, once per month, 2–3 times per month, about once per week, and almost every day. The question about the type of UI asked, “When you lose urine, what is the usual cause?” Responses options were (a) “coughing, sneezing, laughing or doing physical activity” (stress); (b) “a sudden urgent need to go to the bathroom” (urge); “Both (a) and (b) equally” (mixed); and “In other circumstances” (other). Women reporting “other” were asked to specify: responses included “waiting too long to go to the bathroom”, “taking diuretics”, and “drinking too many fluids”. We assessed self-reported UI among the 1091 women who completed a duplicate questionnaire during the 2011 follow-up cycle. Eighty-three percent of women reported a frequency of less than monthly, and 77% of women reporting at least weekly leakage answered the second questionnaire within 1 category of their original response.

#### Covariates.

Data on potential confounders were obtained from the same (or prior) questionnaire on which gestational diabetes was reported. Otherwise, the variables were obtained from the 2011 questionnaire when data on UI were reported. These include age (years), current weight (pounds), vigorous physical activity, smoking, parity, and diuretic use. Self-reported adult height (feet and inches) was collected at baseline (1995). Completed education (≤12, 13–15, ≥16 years) was obtained in 1995 and updated in 2003. Body mass index (BMI) (not during pregnancy) was calculated for the 2011 questionnaire cycle as kg/m^2^. Information was also collected on dietary intake in 2001 using the short-form National Cancer Institute-Block Food Frequency Questionnaire (46); from these data, we calculated prudent (high in fruits/vegetables) and Western (high in meat/fried foods) dietary patterns (47). A neighborhood socioeconomic status (NSES) score was derived from socioeconomic data obtained by linking the women’s 2011 residential addresses to U.S. Census block-group data on wealth, income, and education (48, 49).

#### Data Analysis.

We estimated odds ratios (OR) and 95% confidence intervals (95% CI) for the association between GD or T2D (separately) and UI using multivariable-adjusted logistic regression models (PROC LOGISTIC, SAS version 9.4, SAS Institute, Inc., Cary, NC, USA). The age-adjusted model included terms for age (years), while the multivariable-adjusted model included age plus BMI (<25, 25–29, 30–34, ≥35 kg/m2), parity (1, 2, ≥3), completed education (12, 13–15, ≥16 years), NSES (quintiles, 1 = low, 5 = high), Western dietary patterns (quintiles, 1 = low, 5 = high), prudent dietary patterns (quintiles, 1 = low, 5 = high), vigorous physical activity (none, <5 years, ≥5 h/week), cigarette smoking (current, past, never), and diuretic use (yes, no). Indicator variables were used where data were missing. We stratified analyses according to T2D status (ever, never) in order to explore the GD/UI association independent from and in combination with T2D.

## 3. Results

A total of 1611 and 4514 women, respectively, reported a diagnosis of GD and T2D through 2009. The mean age of the analytic sample was 24.0 (SD = 6.2). UI was common, with more than 50% of women reporting some frequency. Most covariates were associated with UI frequency (Table 1).

BMI, ≥3 births, Western diet (Q5), smoking, and diuretic use were positively associated with increased UI frequency, while vigorous physical activity was inversely associated. Overall, there were no clear associations between baseline characteristics and UI subtypes. Only diuretic use showed a positive association with both urge and mixed incontinence.

Table 2 provides the odds ratios for the association of GD and T2D, respectively, with UI frequency. The age- and multivariable-adjusted ORs for women with a history of GD, compared to those without, were 1.48 (95% CI: 1.25, 1.75) and 1.36 (1.15, 1.62), respectively, for UI frequency of 2–3 times per month, and 1.32 (1.14, 1.52) and 1.18 (1.02, 1.37), respectively, for weekly or greater frequency of UI. For women with a history of T2D, compared to those without, the corresponding age- and multivariable-adjusted ORs for 2–3 monthly UI episodes were 1.44 (1.28, 1.61) and 1.15 (1.02, 1.30), while for those with weekly or more instances of UI, the values were 1.68 (1.54, 1.83) and 1.16 (1.06, 1.27).

We assessed the relationship between GD and T2D and UI subtypes (Table 2). For GD, there was little difference between age- and multivariable models across phenotypes. The multivariable-adjusted ORs for stress and mixed UI among women with GD compared to women without were 1.18 (1.03, 1.36) and 1.31 (1.12, 1.54), respectively. The multivariable-adjusted ORs for the association of T2D with urge and mixed UI were 1.20 (1.10, 1.31) and 1.28 (1.16, 1.42), respectively. There was no clear association between either GD or T2D and “other” UI, although the estimate was stronger for women with GD.

**Figure 2.**
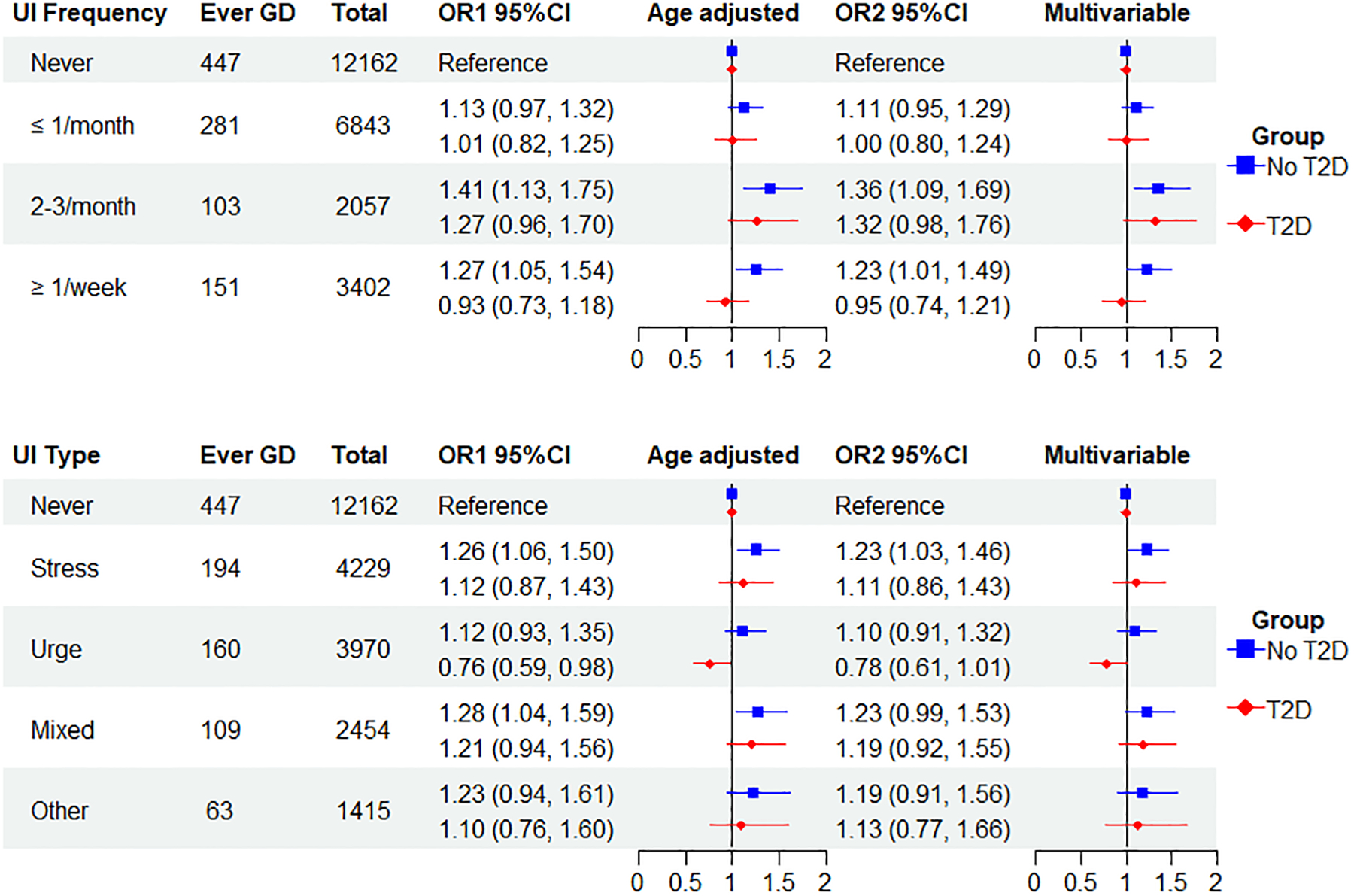
Odds ratios (ORs) of gestational diabetes (GD) and urinary incontinence frequency and type stratified by type 2 diabetes (T2D) status in parous women with and without GD.

Figure 2 presents the analyses of GD and UI frequency stratified by a history of T2D (no T2D, T2D). Overall, the associations between GD and UI frequency were stronger among women without T2D (GD alone) than among those with T2D. The multivariable-adjusted OR for the GD and UI association among those without T2D reporting 2–3 times/month and weekly or more UI was 1.36 (1.09, 1.69) and 1.23 (1.01, 1.49), respectively. Among those with a history of T2D, the respective ORs were 1.32 (0.98, 1.76) and 0.95 (0.74, 1.21). We found a similar pattern for UI subtypes, as stress and urge UI multivariable estimates were stronger among women without T2D. In contrast, the multivariable ORs for mixed and other UI were similar in both strata of T2D.

## 4. Discussion

In this study of nearly 29,000 Black women in the U.S., GD was positively associated with UI after controlling for several potential confounders. Women with GD, even in the absence of T2D, were 36% and 23% more likely to report UI at a frequency of 2–3 times monthly and ≥1/week occurrences, respectively. These findings suggest that women who experience GD—even without subsequent development of T2D—may be at increased risk of UI. We also explored the relationship between T2D status and UI frequency and found positive, albeit weaker, associations with UI frequency 2–3 times/month and weekly or more, consistent with previously reported findings (14, 16, 20–22). The relationships between both GD and T2D with UI subtypes were less clear. Overall, both conditions were modestly associated with increased risk of stress, urge, and/or mixed UI, but these associations did not always achieve statistical significance.

T2D has previously been associated with an increased risk of UI (24, 50). In contrast, GD has received relatively little attention as an independent risk factor for UI. Black women with a history of GD have an increased risk of subsequent development of T2D compared to White women (11, 21, 32, 33). Nevertheless, studies of GD and UI, to date, have involved mostly White, Asian, and non-U.S. populations (20, 21, 36, 37). Our findings are consistent with several analyses linking states of hyperglycemia (GD, T2D, and impaired fasting glucose (IFG)) to UI. For example, in a study of women with GD enrolled in a managed care plan, nearly half of the participants reported stress UI at least weekly during pregnancy and postpartum (36). In other analyses, the association between GD and UI was observed among women undergoing cesarean births, where the presumed adverse effects of vaginal birth on the pelvic floor had been avoided (38, 39).

Approximately 43 million adults are estimated to have “pre-diabetes”, or IFG (51, 52). Studies have found correlations between pre-diabetes, for which GD may be a surrogate measure, and UI (14, 20, 36, 37). A cross-sectional analysis by Brown et al. (20) estimated the prevalence of UI among 1461 nonpregnant adult women in the 2001–2002 National Health and Nutrition Examination Survey (NHANES) to be 35.4% among those with T2D. Similarly, the prevalence among those with IFG was 33.4%. The prevalence of UI among those with normal fasting glucose levels was 16.8%, approximately half of that observed in the two hyperglycemic groups (20). In a more recent study of over 10,000 women included in the 2007–2016 NHANES dataset, investigators found a positive association between pre-diabetic HbA1c levels and prevalence and frequency of SUI (53). Forty percent of the study sample reported SUI (n = 4305), of which 32% had pre-diabetic HbA1c levels ranging from 5.7 to 6.5, compared to 25.9% among those without SUI. Furthermore, approximately 9% of the study sample reported experiencing SUI weekly, of which 38.5% were classified as pre-diabetic (53). These findings lend further support for the association between hyperglycemia and UI.

The exact pathophysiology linking GD with UI is not clear. Both animal and human studies suggest that the hypoglycemic environment impacts the structure and morphology of skeletal muscle tissue, leading to atrophy and impairment of muscle function (34). It has also been suggested that such changes may similarly impair pelvic floor muscle function (35). Hyperglycemia, however, has been associated with an increased urine volume and over-activity of bladder smooth muscle (54), both key factors in UI and urgency UI in particular (21, 55). For example, hyperglycemia can cause osmotic diuresis, increasing urinary frequency and amplifying the risk of incontinence (21). Patients with diabetes are also at increased risk of urinary tract infections, which may exacerbate existing lower urinary tract symptoms, including increased urinary frequency and incontinence (56). Further, microvascular damage associated with hyperglycemia includes alteration of the detrusor smooth muscle and the nerves of the bladder or sphincter muscles, resulting in urethral dysfunction and involuntary bladder contractions (14, 54, 57).

Hyperglycemia may also impact muscles beyond the pelvis, such as abdominal muscles, which are implicated in the function of pelvic floor muscles (58) and urethral closure (59). Results from a study by Catinelli et al., using rat models with mild hyperglycemia during pregnancy, suggest that atrophy of both the rectus abdominis and pelvic floor muscles results from a shift in maternal fiber type composition and increased collagen deposition (60). A separate study, however, compared the impact of diabetic pregnancy on the rectus abdominis muscle and found an increase in the number of slow fibers, possibly indicating a change in the functionality of skeletal muscles exposed to excess glucose (34). Finally, a cross-sectional study evaluating pregnant women from 28 weeks gestation found that GD, lower levels of pelvic floor muscle strength, and UI, were associated with significantly lower levels of relaxin, a reproductive hormone thought to play a role in maintaining urinary continence by degrading collagen in pelvic floor connective tissue, loosening the muscle to facilitate delivery (35). Thus, lower levels of relaxin may be associated with higher levels of fibrosis and subsequent urinary tract dysfunction (35). Previous studies also show that diabetes is characterized by an increase in muscle collagen (61, 62). Despite these observations, the link, if any, between GD and myopathy remains largely unexplored and without an effective treatment (60).

Overall, we found associations between both T2D and GD and all UI subtypes, although we did not observe a significant difference across phenotypes. We found increased associations between T2D and both urge and mixed UI. In a study of the predominantly White Nurses’ Health Study I and II cohorts, Danforth and colleagues found that T2D was associated with a 40% increased odds of at least weekly episodes of urge incontinence: 1.4 (1.0, 1.9) (21). Studies have also reported UI phenotypes according to race. Data from the Study of Women’s Health Across the Nation (SWAN) showed an increased prevalence of urgency UI in Black women (n = 719; 85 cases) compared to other racial groups (13). Similar results were reported by Townsend et al., where urgency incontinence was the most common subtype reported among Black women in the Nurses’ Health Study cohorts (n = 1138; 19 cases) (12).

Our analysis also has several limitations. Because we asked about any history of UI in 2011, after we collected data on GD, we were unable to establish the temporal sequence between GD and UI. We relied on self-reported GD without medical record confirmation, but found high agreement in duplicate questionnaires. Because GD is diagnosed by the end of the second trimester and intensively managed during the remainder of pregnancy, affected women are likely to remember having been diagnosed (63). Validation of maternal self-report of GD against perinatal records in the CHARGE (Childhood Autism Risks from Genetics and the Environment) study reported 70% to 85% for sensitivity and ≥98% for specificity (63). The New York State Pregnancy Risk Assessment Monitoring System (PRAMS) survey estimated a prevalence- and bias-adjusted kappa of 0.88 for 258 births, indicating very good agreement between self-report and birth records for GD (64). Our data suggested acceptable reliability of self-reported UI. Several studies have evaluated self-reported UI versus a detailed physical exam and found high validity of the self-report. Diokno et al. (65) reported an 86.5% agreement between self-reported and clinically diagnosed UI among 169 women aged 60 and older. In a larger group of 456 women from the MESA study, Herzog and Fultz (66), collected UI information both through a self-reported questionnaire and a clinical exam, finding an 83% agreement between the two methods. The results of our validation are consistent with these findings. Furthermore, we used instruments applied to other studies. Finally, the BWHS is not a probability sample of Black women in the U.S. Participants have higher educational status, underrepresenting the 15% of American Black women nationally who have not completed high school (67). Conversely, participants reside in all regions of the United States. Thus, the present results might apply to a large population of Black women in the U.S. The current study also has several strengths, including the large sample size and the successful follow-up of the cohort. To our knowledge, this is the largest study to date to explore the role of GD in relation to UI risk in American Black women. We controlled for many factors of relevance to UI occurrence in multivariable analyses, including parity and BMI, which are established risk factors for UI and are also associated with GD. In addition, our validation of T2D showed high accuracy of self-report. Thus, the estimated prevalence of undiagnosed T2D in the cohort would likely have had a small effect on the risk estimation (68).

## 5. Conclusions

Urinary incontinence is a condition that can greatly impact the quality of life and finances of those affected. Our findings suggest that gestational diabetes may be an independent risk factor for UI in American Black women, challenging the narrative that the effects of GD resolve soon after delivery. Thus, it may be beneficial to create additional screening paradigms for UI for postpartum women with a history of GD. This would allow the practitioners who care for them to initiate earlier interventions such as pelvic physical therapy or expert consultation with a urogynocologist or urologist. Our study is an important first step and will contribute to the identification of younger, high-risk women who may benefit from earlier intervention, management, and treatment of UI.

## Figures and Tables

**Table 1.** Age-standardized characteristics by urinary incontinence frequency and type among parous women, BWHS (N = 28,978).

	Frequency of Urinary Incontinence				Type of Urinary Incontinence (Any Frequency)^a^			
	Never (n = 14,045)	≤1/Month (n = 8087)	2–3 Month (n = 2523)	≥1 Week (n = 4323)	Stress (n = 4946)	Urgency (n = 4886)	Mixed (n = 3123)	Other (n = 1680)
Age, years, mean (SD)	24.2 (6.2)	24.1 (6.2)	23.8 (6.2)	23.2 (6.0)	24.3 (6.2)	24.4 (6.3)	23.8 (6.1)	23.0 (6.0)
Body Mass Index, kg/m^2^, mean (SD)	27.3 (5.9)	28.1 (6.2)	29.2 (6.8)	30.2 (7.0)	27.3 (5.9)	28.1 (6.0)	29.0 (6.7)	30.0 (7.0)
Parity (%)								
1 birth	47	44	43	40	44	41	42	43
2 births	30	32	32	31	33	32	31	29
3+ births	23	24	25	29	23	27	27	27
Years of education (%)								
≤12	19	17	19	20	17	18	20	16
13–15	36	36	37	36	37	35	37	37
≥16	44	47	44	44	46	47	43	47
Neighborhood socioeconomic status, quintiles (%)								
Q1 (low)	18	18	19	18	17	18	20	19
Q5 (high)	19	19	18	18	20	19	16	18
Western diet, quintiles (%)								
Q1 (low)	20	19	17	17	17	18	16	21
Q5 (high)	17	19	18	21	19	20	21	17
Prudent diet, quintiles (%)								
Q1 (low)	19	18	19	18	19	17	19	18
Q5 (high)	19	18	18	18	17	20	16	19
Vigorous activity, hours/week (%)								
None	35	34	36	42	34	38	38	35
<5	49	52	49	45	52	48	47	51
≥5	11	10	10	9	10	9	10	10
Cigarette smoking status, (%)								
Current	13	13	15	17	13	15	16	13
past	21	22	23	25	21	23	24	25
never	66	65	62	58	65	62	60	62
Recent diuretic use (2011), (%)								
Yes	10	11	13	15	9	14	14	12

a A total of 298 women who reported a frequency of urinary incontinence did not report a type of urinary incontinence. Values are means (SD) or percentages and are standardized to the age distribution of the study population. Unless otherwise indicated, variables were obtained from the same (or prior) questionnaire on which gestational diabetes was reported. Otherwise, the variables were obtained from the 2011 questionnaire when data on UI were reported. Some frequencies may not sum to 100% due to missing values.

**Table 2.** Odds ratios (ORs) of gestational diabetes (GD), type 2 diabetes (T2D), and urinary incontinence frequency and type in parous women, BWHS (N = 28,978).

			Urinary Incontinence Frequency			Urinary Incontinence Type			
			OR 95% CI^a^			OR (95% CI)^a^			
Ever Gestational Diabetes^b,c^		Never	≤1/month	2–3/month	≥1 week	Stress	Urge	Mixed	Other^d^
n	Yes	707	457	179	268	304	262	212	103
	Total	14,045	8087	2523	4323	4946	4886	3123	1680
Age-Adjusted		REF	1.14 (1.01, 1.29)	1.48 (1.25, 1.75)	1.32 (1.14, 1.52)	1.24 (1.08, 1.43)	1.10 (0.95, 1.27)	1.48 (1.25, 1.72)	1.24 (1.00, 1.54)
Multivariable-Adjusted^e^		REF	1.09 (0.97, 1.23)	1.36 (1.15, 1.62)	1.18 (1.02, 1.37)	1.18 (1.03, 1.36)	1.03 (0.88, 1.19)	1.31 (1.12, 1.54)	1.16 (0.94, 1.44)
Ever Type 2 Diabetes^b,f^									
n	Yes	1883	1244	466	921	717	916	669	265
	Total	14,045	8087	2523	4323	4946	4886	3123	1680
Age-Adjusted		REF	1.17 (1.08, 1.26)	1.44 (1.28, 1.61)	1.68 (1.54, 1.83)	1.09 (1.00, 1.20)	1.46 (1.34, 1.60)	1.68 (1.52, 1.85)	1.20 (1.05, 1.38)
Multivariable-Adjusted^e^		REF	1.07 (0.99, 1.16)	1.15 (1.02, 1.30)	1.16 (1.06, 1.27)	1.02 (0.92, 1.12)	1.16 (1.06, 1.28)	1.23 (1.11, 1.36)	0.99 (0.85, 1.14)

a REF = Reference = never urinary incontinence;

b parous women;

c reference = never having had gestational diabetes.

d Includes situations such as “waiting too long to go to the bathroom”, “taking diuretics”, and “drinking too many fluids”.

e Adjusted for age, BMI, parity, education, NSES, prudent and Western diet, use of diuretics, vigorous physical activity, and smoking status.

f Reference = never having had TD2.

## Data Availability

Data underlying the study cannot be made publicly available due to ethical concerns about patient confidentiality. Data will be made available to qualified researchers on request to BWHS@bu.edu.

## Abbreviations

The following abbreviations are used in this manuscript.

BMI: Body mass index
BWHS: Black Women’s Health Study
GD: Gestational diabetes
HbA1c: Hemoglobin A1c
IFG: Impaired fasting glucose
NHW: Non-Hispanic White
NSES: Neighborhood socioeconomic status
SUI: Stress urinary incontinence
T2D: Type 2 diabetes
UI: Urinary incontinence
US: United States
